# Estimating the impact of the RTS,S/AS01 malaria vaccine in Benin: A mathematical modelling study

**DOI:** 10.1371/journal.pgph.0005543

**Published:** 2026-05-08

**Authors:** Sena V. Alohoutade, Rachel Hounsell, Codjo Dandonougbo, Rock Aikpon, Jules Degila, Sheetal Silal

**Affiliations:** 1 Modelling and Simulation Hub, Africa, Department of Statistical Sciences, University of Cape Town, Cape Town, South Africa; 2 Centre for Global Health Research, Nuffield Department of Medicine, Oxford University, Oxford, United Kingdom; 3 National Malaria Control Program, Ministry of Health, Cotonou, Benin; 4 Institut de Mathématiques et de Science Physiques, Dangbo, Benin; PLOS: Public Library of Science, UNITED STATES OF AMERICA

## Abstract

Malaria constitutes a major public health burden in sub-Saharan Africa. It remains a key health concern and the leading cause of death in children under five in Benin. Since October 2021, the World Health Organization has recommended the use of malaria vaccines for the prevention of *Plasmodium falciparum* malaria in children living in malaria endemic areas, prioritizing areas of moderate and high transmission in sub-Saharan Africa. However, with the exception of Ghana, there is a scarcity of studies modelling the potential impact of the RTS,S/AS01 vaccine in the context of West Africa. A compartmental mathematical model was developed to estimate clinical and severe malaria cases averted in children under five with the primary series of the RTS,S/AS01 malaria vaccine in Benin. Over a period of 10 years, scenarios involving vaccine introduction at different coverage levels alongside the use of long-lasting insecticidal nets were modelled to assess the impact of the RTS,S/AS01 vaccine on malaria transmission. The combination of childhood malaria vaccination at a coverage similar to that of the third dose of diphtheria, tetanus, and pertussis (DTP3) vaccine, along with the current use of nets, is projected to result in a 40% reduction in malaria clinical cases and deaths among children under five years old, compared to using nets alone, from 2025 to 2034. However, if the introduction of a malaria vaccine has the unintended consequence of decreased net use, cumulative benefits may be offset. A 1.5-fold decrease in the use of nets is projected to result in an increase in malaria burden. This modelling exercise concludes that childhood vaccination is expected to avert clinical and severe cases of malaria and is an additional tool to advance malaria control efforts in Benin but potential unintended consequences of a reduction in net usage may reduce these gains.

## Author summary

This study addresses a key research gap by modelling the impact of childhood malaria vaccination on *P. falciparum* malaria transmission in Benin. The findings suggest that combining vaccination at DTP3 coverage levels with existing long-lasting insecticidal net (LLIN) use could reduce malaria cases and deaths among children under five by approximately 40% between 2025 and 2034, compared to using nets alone. However, if vaccine introduction leads to reduced net use, the cumulative benefits may be offset. These results highlight the importance of maintaining high LLIN coverage and use alongside vaccination, and the need for continued engagement with donors.

## Introduction

Malaria constitutes a major public health burden in sub-Saharan Africa. In 2023, the World Health Organization (WHO) reported 263 million cases and 597 thousand deaths globally due to malaria. Africa accounted for 94% of these cases and 95% of the deaths [1]. Two groups are particularly vulnerable to severe malaria infection: children under the age of five and expectant mothers. Children under five accounted for approximately 76% of all malaria deaths in the region [2]. Between 2015 and 2022, malaria incidence remained relatively stable [1].

Benin is one of the fifteen countries with the highest burden of malaria; it accounted for 2% and 1.7% of malaria cases and deaths worldwide in 2023 [1]. Malaria is the leading cause of medical consultation and hospitalization in Benin. It accounts for 48% of medical consultations for children under five years old (CU5) and for 23.1% of deaths recorded in health facilities [3]. The disease exerts an economic strain on the country’s development, as Beninese households allocate approximately a quarter of their yearly income to treating and preventing malaria [4]. Despite extensive attempts to control and eliminate the disease, malaria continues to have a substantial impact on the health and well-being of millions of people across Africa.

Malaria preventive interventions include insecticide residual spraying, the use of bed nets and prophylactic treatment of pregnant women, infants, and children [5]. However, these interventions are not exempt from challenges posed by insecticide-resistance vectors and drug-resistance parasites. Therefore, among the various strategies employed to tackle malaria, vaccination has emerged as a promising approach to complement existing preventive and treatment measures.

RTS,S/AS01_E_ (RTS,S) was the first malaria vaccine recommended by the WHO in October 2021 for use in children living in sub-Saharan Africa and other regions with moderate to high *Plasmodium falciparum* transmission [6]. A second malaria vaccine, R21/Matrix-M, was later recommended in October 2023. The RTS,S vaccine took over 30 years to be developed, approved, and to enter pilot implementation studies [7]. In a pivotal Phase III trial, four doses of RTS,S administered to children of five to seventeen months of age resulted in a 36% reduction in clinical malaria and a 32% reduction in severe malaria, sustained over a four-year follow-up period [8]. Following this trial, RTS,S underwent evaluation in Ghana, Malawi, and Kenya as part of a widespread WHO pilot programme [9].

In April 2024, Benin introduced the RTS,S malaria vaccine into its Expanded Programme on Immunization (EPI), marking a significant milestone in the country’s malaria control efforts. This followed Benin’s strong engagement with Gavi, the Vaccine Alliance, through which it received a total of 215,900 RTS,S doses to support the rollout of the vaccine [10]. In the early phases of this rollout, there remains a need to model the impact and cost effectiveness of vaccination to inform its implementation in Benin and in African countries that have shown interest in its introduction.

Mathematical models of malaria transmission provide valuable evidence to support decision-making, including identifying effective intervention combinations, setting realistic coverage targets, anticipating the impact of new interventions, and assessing the risk of malaria resurgence [11]. Before the recommendation of RTS,S by the WHO, many modelling studies explored the potential impact of its implementation in sub-Saharan Africa [7,12,13].

WHO working group used four mathematical models to assess the health impact and cost effectiveness of the RTS,S vaccine in an African setting [14–17]. Building on this, Penny et al. [18] conducted a comparative analysis of the four malaria models and projected the public health impact of the RTS,S malaria vaccine across diverse transmission contexts. Their study showed that the vaccine’s impact varies by transmission intensity and seasonality, with the greatest reductions in incidence projected in highly seasonal settings such as those found in West Africa. The analysis also indicated that RTS,S would be cost-effective if integrated into existing routine immunization programmes. Similarly, Winskill et al. [13] highlighted that, while scaling up core vector control and treatment interventions should remain a priority, the RTS,S vaccine offers additional benefits in seasonal malaria settings, particularly when very high coverage of LLINs and, where appropriate, seasonal malaria chemoprevention (SMC) has been achieved. Hogan et al. [19], further found that in high-endemic areas, adopting vaccine coverage similar to that of the third dose of diphtheria, tetanus, and pertussis (DTP3) vaccine, could prevent 4.3 million malaria cases and 22,000 deaths in CU5 each year across sub-Saharan Africa.

Taken together, these findings provide useful insights for West African contexts, despite most studies not focusing specifically on Benin. However, assumptions regarding health-care infrastructure, vaccination schedules, and program costs vary widely between low- and middle-income countries (LMICs). As a result, national policymakers increasingly seek country-specific modelling evidence to guide decision-making [12].

Nevertheless, few studies have explicitly modelled the potential impact of RTS,S in West Africa, and only a limited number have assessed its impact alongside existing interventions such as LLINs [20].

Although Benin has recently received initial RTS,S vaccine doses through GAVI, financial and operational challenges continue to impede malaria control efforts in many sub-Saharan African countries. These challenges arise from insufficient resources to support malaria vaccination campaigns, gaps in surveillance systems, delays in data collection, and the limited availability of accurate, region-specific data on malaria transmission dynamics [21,22]. Collectively, these factors contribute to the relatively low number of research publications emerging from the West African region, with fewer studies led by West African researchers within the region [23].

In this study, a compartmental model is employed to project the relative reduction in clinical and severe malaria cases achieved by introducing the RTS,S malaria vaccine in relation to the distribution and use of LLINs in Benin. This will not only provide an assessment of the additive nature of the two interventions but also provide insight into the impact of social behavioural change communication (SBCC) and vaccination campaigns. The impact of SBCC is represented through its effect on the uptake and use of interventions. Therefore, its impact is assessed in the model by varying the coverage levels of these interventions (e.g., increasing LLIN usage or vaccination coverage).

## Methods

### Ethics statement

This study was approved by the Faculty Research Ethics Committee of the University of Cape Town (Reference STU-IFHREC-2025-PSQ001696).

### Study setting

The Republic of Benin is located in West Africa with an estimated population of 14.6 million in 2024. The entire population is at risk of malaria. In Benin, malaria incidence is 212 per 1,000 population at risk and the death rate in CU5 is 96 per 1,000 [24]. In children aged 6–59 months, malaria prevalence increased from about 28% in 2012 to 39% in 2018 and decreased to 32.3% in 2022, with the burden consistently higher in the northern provinces compared to the south, where prevalence exceeds 40% in the north and remains below 30% in the south [3,24]. Malaria transmission is variable across Benin with seasonal and geographic fluctuations closely correlated with rainfall patterns, climate, and topography. The geography and climate provide a favourable environment for malaria persistence [3].

Several interventions have been implemented to prevent malaria in Benin. These interventions include the use of LLINs, SMC and intermittent preventive treatment of malaria during pregnancy (IPTp). The primary pillar of Benin’s vector control strategy is LLINs [25]. Insecticidal treated nets were introduced in the early 2000s at a small scale, with the first nationwide free ITN distribution campaign, targeting children under five and pregnant women, carried out in 2007. The country implemented a universal coverage campaign, aiming to provide one net for every two people in households in 2011. Since then, Benin has continued periodic mass distribution campaigns (roughly every three years) and continuous distribution through antenatal clinics and child health visits [24]. However, the use of nets has increased from 15% in 2006 to 71% in 2018 but decrease to approximately 60% in 2022 [26].

Benin introduced SMC in 2019 in children aged three to 59 months in two northern health zones. Following positive outcomes, including 97% coverage among the target group of children and a subsequent 45% decrease in malaria incidence, the programme was expanded to two additional health zones in Alibori province, in 2020, and further extended to two more health zones in Atacora province, in 2021 [27].

### Data description

Population data were sourced from the United Nations World Population Prospects, facilitating projections of population size, growth, and estimated numbers of individuals for each year of life, relying on fertility and mortality data. We used country-specific coverage for DTP3 vaccination from the WHO/UNICEF Estimates of National Immunization Coverage (WUENIC) as a proxy for RTS,S vaccine coverage [28]. Estimated annual clinical malaria cases, deaths and historical LLINs coverage estimates for Benin were obtained from the World Malaria Report (WMR) annexes [1]. Data from national Demographic and Health Surveys (DHS) and Malaria Indicator Surveys (MIS), which provide cross-sectional estimates of household LLIN ownership and use, were employed to validate and contextualize the LLIN coverage trends reported in the WMR. In the model, LLIN deployment was initiated in 2006, consistent with DHS data. Similarly, assumptions regarding treatment-seeking behaviour were informed by DHS estimates, ensuring that modelled access to treatment reflected country-specific patterns.

### Description of the model

The model is a deterministic age-structured dynamic compartmental model accounting for LLIN use across all age groups and seasonality to estimate the impact of the RTS,S malaria vaccine on clinical malaria cases and deaths in children in Benin. The model explicitly incorporated LLIN use across all age groups, reflecting their role as the primary nationally scaled malaria control intervention in Benin. LLINs are assumed to prevent biting by *Anopheles* mosquitoes when people are under a net. As such, LLINs in our model reduce the effective biting rate of mosquitoes and thereby lower the effective force of infection.

While SMC could be incorporated, its implementation has been restricted to selected health zones (6 out of 34), as SMC was introduced in two health zones in 2019 and expanded gradually to six health zones in 2021. We therefore focused on LLINs at the national level to assess the potential impact of introducing the RTS,S vaccine in Benin at a population-wide scale. Building on this, future sub-national modelling work will explicitly incorporate SMC alongside vaccination to evaluate the combined effects of these interventions in high-burden health zones.

As the burden of malaria varies by age, the model considered the age structure of the population, determined by both the vaccination schedule and the age profile for the severity of the disease. According to a large phase III trial that included several endemic areas of Africa, the RTS,S vaccine efficacy against clinical malaria begins at 74% in children aged five to 17 months, a few weeks after the last immunization, and drops to 9% after five years [29].

In Ghana, the vaccine is administered to children in four doses, beginning at six months, followed by doses at seven, nine, and 18 months [30]. Following the 215,900 doses of the malaria vaccine acquired in January 2024, Benin has begun administering since April 2024, three doses to children at six, seven, nine months and a fourth dose as a booster for children at 18 months of age [10]. In this primary analysis, we modelled only the first three doses using a single-phase exponential decay with an average duration of four years, consistent with previous malaria transmission models [31]. We assumed that children received the three doses at six, seven, and nine months. The assumptions also include the fact that all the vaccinated children receive the three doses of the vaccine. In this regard, any individual that received the first dose get all the three doses as studies have only estimated efficacy following the full three-dose primary series. The model does not account for vaccine efficacy before the third dose.

**Fig 1:**
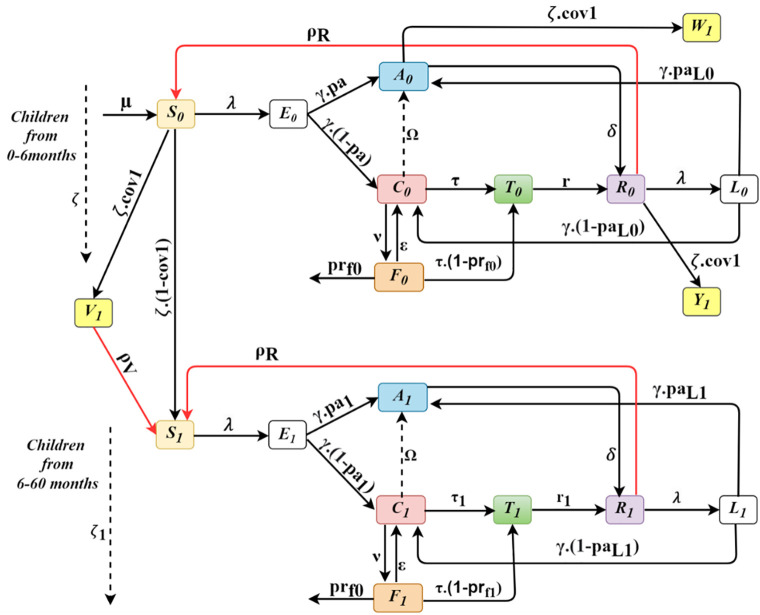
Compartmental model showing the first two age groups with compartments Si (Susceptible), E_i_ (Exposed), A_i_ (Asymptomatic), C_i_ (Symptomatic), T_i_ (Treated), R_i_ (Recovered), L_i_ (Second Exposure) and V_i_, W_i_,Y_i_ (Vaccination compartments).

The model categorizes the human population into three age groups: children from birth to six months, those aged six months to five years, and individuals aged five years and older (Fig 1). Susceptible individuals are exposed to infectious bites at a rate depending on mosquito density and infectivity. Although mosquito biting can vary with age, high LLIN coverage, consistent net use, and indoor sleeping are expected to make exposure similar across age groups [32]. We therefore assume homogeneous biting, while age-specific differences in malaria outcomes are captured through immunity development. Following exposure (E_i_), there is a latent period after which the infected individuals may experience an asymptomatic (A_i_) episode of malaria according to their immunity level or develop clinical symptoms (C_i_). The chance of symptomatic disease decreases with continued exposure due to the development of naturally acquired immunity. Asymptomatic individuals do not display symptoms but can transmit parasites to mosquitoes at a rate less than the rate at which symptomatic individuals transmit gametocytes (Table B in S1 Appendix). However, asymptomatic individuals recover naturally after a period of time. A clinical episode, where individuals are symptomatic, can be uncomplicated (C_i_) or progress to a severe malaria episode (F_i_) depending on the immunity levels, with children being more prone to developing severe infection as they have less immunity compared to adults. A severe malaria episode can result in death. Symptomatic individuals are destined to be treated (T_i_), after which they recover (R_i_). Recovered individuals (Ri) are assumed to gain natural immunity that provides temporary protection against developing a new malaria episode. This natural immunity wanes over time, after which individuals return to the susceptible compartment (R → S).

We consider a natural mortality rate from each compartment. Additionally, malaria-related directly-attributable deaths are explicitly considered in the severe stage of modelled infection. We assume that children transition between age groups at age-specific rates (ζ₁, ζ₂) that reflect the durations of each group. Specifically, infants move from zero to six months age group to six months to five years group at rate ζ₁, while children move from six months to five years to five plus years at rate ζ₂. This approach accounts for the different sizes and time spans of the three age groups in the model. In our model, children become eligible for vaccination starting at six months of age; however, vaccine-induced protection is only assumed to take effect after completion of the full three-dose schedule, with the third dose administered at nine months. Vaccinated individuals acquire vaccine-induced immunity that prevents them from developing clinical malaria. As vaccine-induced protection is not permanent, vaccinated individuals might lose the immunity due to waning after four years, resulting in individuals becoming susceptible again (V → S), as shown in Fig 1. Vaccine impact is therefore estimated for children under five following completion of the three-dose schedule, not immediately at six months.

Fig 1 shows a diagram of the compartmental model showing the first two age groups.

Prior to the introduction of RTS,S vaccination, the model was simulated over an extended burn-in period under historical intervention coverage to allow the system to reach a stable endemic regime. The model was subsequently calibrated to observed epidemiological data, and vaccine impact was assessed from this calibrated baseline, ensuring that results were not influenced by transient dynamics.

Seasonality is modelled by multiplying the force of infection of the model by a seasonality factor. The average monthly rainfall at a country level was standardized and fitted to a trigonometric function, which was then used as a forcing function to account for seasonality. The full description of the model, calibration, parameters table, and differential equations are available in the supplementary file.

### Scenarios

Six scenarios of vaccine coverage and effective LLIN coverage, classified under two categories, are simulated over a period of 10 years to provide estimates of the malaria burden alleviated by these interventions in CU5.

The baseline represents the existing effective LLIN coverage, assuming that LLIN coverage and use will remain at the average levels for 2020–2022 [33]. The baseline serves as a comparison for the other scenarios.

The first three scenarios model vaccine introduction with no impact on other interventions. The first scenario (76% vaccination + 43.2% effective LLIN coverage) includes the introduction of vaccination at DTP3 coverage level (76%) with no impact on net use and distribution. In this scenario, all eligible children are included in the targeted population; however, the uptake of the vaccine is assumed based on the administration of the third dose of DTP3, using country-level 2022 DTP3 coverage data from the WUENIC [34].

The second scenario (50% vaccination + 43.2% effective LLIN coverage) includes vaccine introduction assuming that a lower level than DTP3 coverage is achieved (50%) with no impact on net use and distribution.

The third scenario (85% vaccination + 43.2% effective LLIN coverage) includes vaccine introduction, assuming a coverage level higher than DTP3 is achieved (85%) with no impact on net use and distribution.

The second set of three scenarios models vaccine introduction with impact on effective LLIN coverage. The fourth scenario (76% vaccination + 28.8% effective LLIN coverage), includes vaccine introduction at DTP3 coverage with a negative impact on effective LLIN coverage. It assumes a vaccine coverage of 76% in children (as in Scenario 1) and a decrease to 28.8% in effective LLIN coverage (from the baseline of 43.2%) to simulate the potential negative impact of vaccine introduction on LLIN use. This scenario is purely hypothetical and considers the possibility that the perceived protection from the vaccine might lead to a reduction in LLIN use among the population. While there is currently no evidence that RTS,S introduction decreases LLIN use, including this scenario allows us to explore the potential consequences of reduced net use under such conditions.

In the fifth scenario (85% vaccination + 50.4% effective LLIN coverage), we considered a social behavioural change communication campaign (SBCC) to improve vaccine coverage and net use. The campaign is modelled to result in vaccine coverage of 85% in the targeted population, and an increase in effective LLIN coverage from baseline (43.2%) to 50.4%.

The sixth scenario (0% vaccination + 28.8% effective LLIN coverage) is considered a reverse scenario, designed to assess the relative contribution of vaccination by comparing outcomes with and without its addition under reduced effective LLIN coverage. It assumes no vaccine introduction (0% coverage in children) and a decline in effective LLIN coverage from 43.2% (baseline) to 28.8%. This reduction could result from either a decrease in net availability due to funding constraints or reduced use resulting from behavioural changes within the population. However, in this scenario, we assume that overall LLIN coverage remains constant and the decline in effective LLIN coverage is a result of reduced net use.

Table 1 presents an overview of the scenarios.

**Table 1:** Scenarios and coverage levels for modelling.

		Scenarios			
Categories	Variables	Baseline	Scenario 1 (DTP3_cov)	Scenario 2 (Low_cov)	Scenario 3 (High_cov)
Vaccine introduction with no impact on other interventions	Vaccine coverage	**–**	76%	50%	85%
	Effective LLIN coverage	2020–2022 effective coverage 43.2% (72% coverage *60% use)			
	**Variables**		**Scenario 4 (DTP3_cov + low LLIN)**	**Scenario 5** Optimistic **(High_cov+high LLIN)**	**Scenario 6** **Reduced LLIN**
Vaccine introduction with impact on other interventions	Vaccine coverage		76%	85%	–
	Effective LLIN coverage		28.8% (72% coverage *40% use)	50.4% (72% coverage *70% use)	28.8% (72% coverage *40% use)

#### Sensitivity analysis

A sensitivity analysis was conducted to evaluate the validity of our results and determine how changes in key parameters influence the projected impact on clinical malaria cases over the 10-year period. A univariate sensitivity analysis was performed on non-vaccine-related parameters (treatment-seeking period, net use, and net effectiveness) using Microsoft Excel version 2508, while a multivariate sensitivity was conducted on vaccine-related parameters (coverage, efficacy, waning) using R software version 4.3.3. The full detail on the sensitivity analysis conducted is available in S1 Appendix.

#### Estimation of uncertainty intervals for model outputs

Uncertainty intervals were estimated by running 200 simulations per model scenario. In each simulation, parameters were randomly sampled from their respective distributions within defined lower and upper bounds (Table B in S1 Appendix). Daily malaria case estimates were then aggregated across all simulations, and the median, along with the 5th and 95th quantiles, was computed for each day.

## Results

Six scenarios, each simulated over a ten-year period (2025–2034), were used to evaluate the impact of varying vaccine coverage levels and effective LLIN coverage on malaria cases and deaths in CU5 and the total population in Benin. The reduction in malaria burden associated with each scenario over the cumulative period from 2025 to 2034 was assessed. This approach highlights the broader impact of vaccination by comparing the projected cases, severe cases, and deaths averted under each scenario to the baseline estimates.

**Fig 2:**
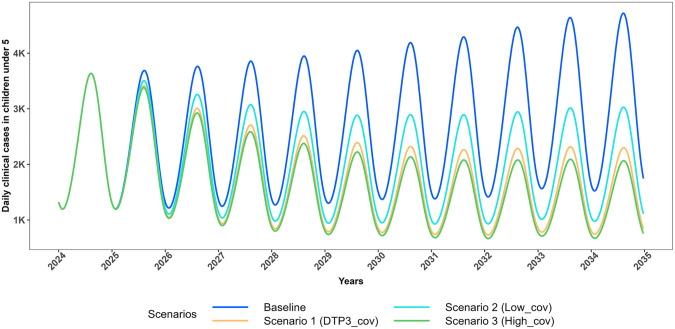
Projected trajectories of *P. falciparum* malaria daily clinical cases in CU5 for scenarios with no change in effective LLIN coverage (2025–2034). Trends in projected daily clinical malaria cases among children under five (CU5) from 2024 to 2035, comparing intervention scenarios assuming no change in effective LLIN coverage (maintained at 43.2%). The Baseline scenario models 0% vaccination coverage (blue). Scenario 1 assumes 76% vaccination coverage with 43.2% effective LLIN coverage (orange); Scenario 2 assumes 50% vaccination coverage with 43.2% effective LLIN coverage (cyan); and Scenario 3 assumes 85% vaccination coverage with 43.2% effective LLIN coverage (green).

**Fig 3:**
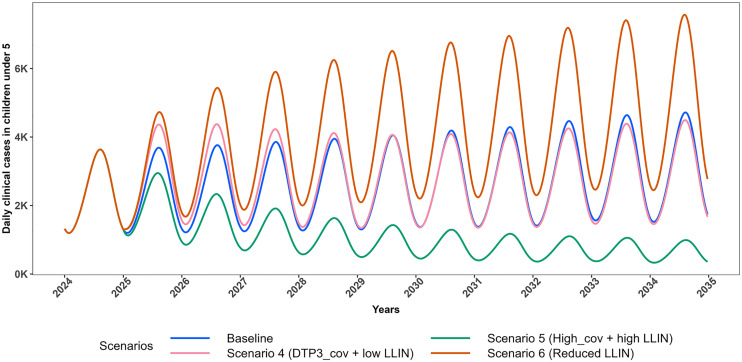
Projected trajectories of *P. falciparum* malaria daily clinical cases in CU5 for scenarios with change in effective LLIN coverage (2025–2034). Trends in projected daily clinical malaria cases among children under five (CU5) from 2024 to 2035, comparing intervention scenarios involving changes in effective LLIN coverage to the baseline. The Baseline scenario assumes 0% vaccination coverage with 43.2% effective LLIN coverage (blue). Scenario 4 assumes 76% vaccination coverage with 28.8% effective LLIN coverage (pink); Scenario 5 assumes 85% vaccination coverage with 50.4% effective LLIN coverage (dark green); and Scenario 6 assumes 0% vaccination coverage with 28.8% effective LLIN coverage (dark orange).

Figs 2 and 3 illustrate the projected trajectories of daily clinical cases in CU5 for Scenarios 1–3 and 4–6 respectively. Scenarios 1–3 simulate vaccine introduction at different coverages with no impact on effective LLIN coverage while, scenarios 4–6 simulate vaccine introduction at different coverages with impact on effective LLIN coverage.

In the baseline scenario, the daily incidence of clinical malaria increases with population growth over time though incidence rate remains relatively stable throughout the simulation period (2025–2034). In Scenario 1, which simulates the introduction of RTS,S vaccine at DTP3 coverage levels, there is a substantial reduction of 40% in clinical malaria cases in CU5 over ten years, equivalent a 1.67-fold reduction in all outcomes compared to the baseline (Table 2–3, Figs 6–7).

**Table 2:** Projected number of cases and deaths in CU5 across the six scenarios compared to the baseline.

In children under 5 years	Number of cases		Number of deaths	
	By 2029	By 2034	By 2029	By 2034
**Baseline**	4,607,600 (3,430,036–5,345,265)	9,959,000 (7,126,937-11,602,994)	32,778 (24,545-38,106)	70,852 (50,864-82,722)
**Scenario 1**	3,368,370 (2,498,528-3,898,248)	6,125,590 (4,176,314-7,188,987)	23,900 (17,880-27,727)	43,440 (29,779-51,093)
**Scenario 2**	3,759,240 (2,788,067-4,356,564)	7,295,030 (5,037,974-8,543,055)	26,700 (19,951-31,013)	51,790 (35,931-60,788)
**Scenario 3**	3,240,840 (2,404,921-3,748,260)	5,756,200 (3,911,767-6,758,685)	22,990 (17,210-26,652)	40,800 (27,891-48,013)
**Scenario 4**	5,107,075 (4,069,786-5,862,869)	10,305,800 (8,080,566-11,882,147)	36,190 (28,991-41,638)	72,980 (57,400-84,340)
**Scenario 5**	2,497,230 (1,808,697-2,897,338)	3,958,510 (2,533,944-4,695,046)	17,740 (12,992-20,625)	28,095 (18,137-33,390)
**Scenario 6**	6,914,240 (5,602,600-7,958,942)	15,559,540 (12,786,807-17,903,668)	49,140 (39,970-56,690)	110,610 (91,087-127,556)

**Table 3:** Projected changes in percentage of cases and deaths in CU5 across the six scenarios compared to the baseline.

In children under 5 years	Percentage of cases		Percentage of deaths	
	By 2029	By 2034	By 2029	By 2034
**Scenario 1**	-27% (16-46)	-40% (28-58)	-28% (15-45)	-40% (28-58)
**Scenario 2**	-19% (6-39)	-27% (14-49)	-19% (6-39)	-27% (14-49)
**Scenario 3**	-30% (19-47)	-43% (32-61)	-30% (19-48)	-43% (32-61)
**Scenario 4**	+10% (-12-21)	+4% (-16-19)	+10% (-12-21)	+3% (-16-18)
**Scenario 5**	-46% (37-61)	-62% (53-75)	-46% (37-60)	-61% (53-74)
**Scenario 6**	+33% (23-50)	+36% (25-54)	+33% (23-50)	+36% (22-50)

Positive values show an increase compared to the baseline and negative values show a decrease compared to the baseline.

Scenario 2, which simulates the introduction of the vaccine at coverage levels lower than DTP3 (50% vs 76% coverage), projected a 27% reduction in clinical cases, severe cases, and deaths in CU5—less than the impact observed in Scenario 1 (Table 2–3, Figs 6–7). This corresponds to a 1.37-fold reduction in all outcomes over ten years. This comparison highlights that a decrease in vaccine coverage, as seen in Scenario 2, undermines the potential health benefits, reinforcing the need to maintain or even enhance vaccine coverage to maximize public health gains.

Scenario 3 simulates the introduction of the vaccine, assuming a coverage level higher than DTP3 is achieved (85% vs 76% coverage) with no impact on effective LLIN coverage. Under this scenario, there is a substantial projected reduction of 43% in clinical malaria cases in CU5 (Table 2–3, Figs 6–7). This highlights the additional health benefits of increasing vaccine coverage, reinforcing the importance of achieving high uptake to maximize malaria burden reduction. The trajectory of *P. falciparum* malaria clinical cases in CU5 for each scenario influences the corresponding trends in severe cases and malaria-related deaths in CU5 (Figs C and D in S1 Appendix).

Scenarios 4–6 were constructed to explore the possible unintended impact of vaccine introduction on other interventions. Scenario 4 simulates vaccine introduction at DTP3 coverage levels combined with a reduction in effective LLIN coverage (from 43.2% to 28.8%). Initially (2025–2029), daily clinical malaria cases rise compared to the baseline in CU5, but from 2030 onward, cases decline (Fig 3). This pattern reflects the vaccine’s age-specific administration (six months to nine months). Initially, the impact is offset by increased malaria cases in unvaccinated children, resulting from a reduction in effective LLIN coverage. However, by 2030, most under-fives are vaccinated, leading to a significant reduction in clinical cases. This scenario projected approximately 4% increase in all outcomes compared to the baseline in CU5 over the 10 year period (Table 2–3, Figs 6–7). Notably, this scenario substantially impacts the total population, resulting in an approximate 20% increase in malaria cases and a 12% rise in malaria-related deaths compared to baseline levels (Fig 8, Figs G and J in S1 Appendix).

This finding indicates that the introduction of vaccination at DTP3 coverage levels, alongside reduced effective LLIN coverage, may mask the vaccine’s impact in CU5, as this scenario closely resembles the baseline over the ten-year period. Overall, the increase in malaria cases is more pronounced in the total population than in CU5, likely because LLINs serve as the main intervention for protection across the entire population, and Scenario 4 involved a reduction in their effective coverage. This highlights the critical role of sustained LLIN distribution and use in maximizing vaccination benefits.

**Fig 4:**
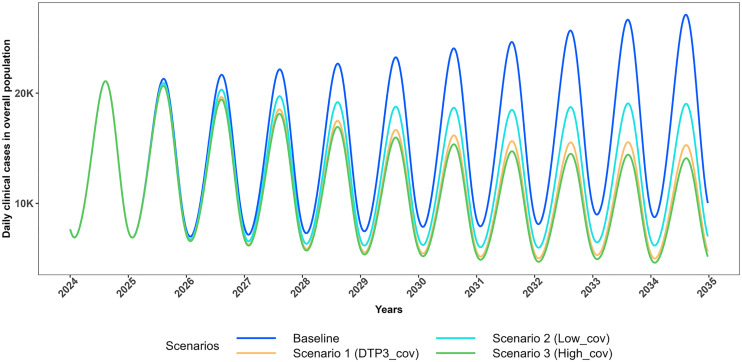
Projected trajectories of *P. falciparum* malaria daily clinical cases in the total population for scenarios with no change in effective LLIN coverage (2025-2034). Trends in daily clinical malaria cases in total population from 2024 to 2035, comparing intervention scenarios assuming no change in effective LLIN coverage (maintained at 43.2%). The Baseline scenario models 0% vaccination coverage (blue). Scenario 1 assumes 76% vaccination coverage with 43.2% effective LLIN coverage (orange); Scenario 2 assumes 50% vaccination coverage with 43.2% effective LLIN coverage (cyan); and Scenario 3 assumes 85% vaccination coverage with 43.2% effective LLIN coverage (green).

**Fig 5:**
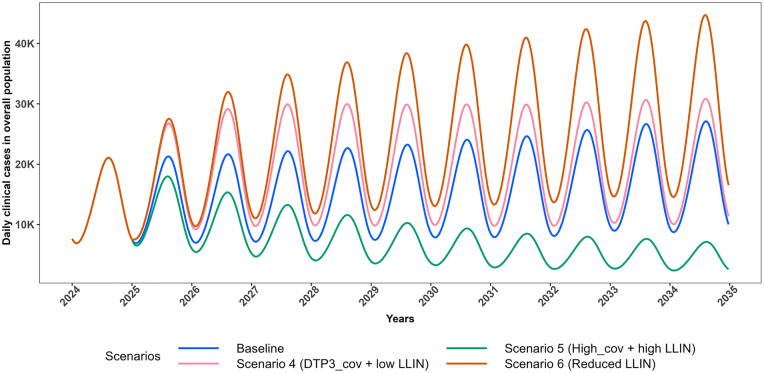
Projected trajectories of *P. falciparum*malaria daily clinical cases in the total population for scenarios with change in effective LLIN coverage (2025–2034). Trends in projected daily clinical malaria cases in total population from 2024 to 2035, comparing intervention scenarios involving changes in effective LLIN coverage to the baseline. The Baseline scenario assumes 0% vaccination coverage with 43.2% effective LLIN coverage (blue). Scenario 4 assumes 76% vaccination coverage with 28.8% effective LLIN coverage (pink); Scenario 5 assumes 85% vaccination coverage with 50.4% effective LLIN coverage (dark green); and Scenario 6 assumes 0% vaccination coverage with 28.8% effective LLIN coverage (dark orange).

Figs 4 and 5 respectively illustrate the projected trajectories of daily clinical cases in the total population for each set of the scenarios.

**Fig 6:**
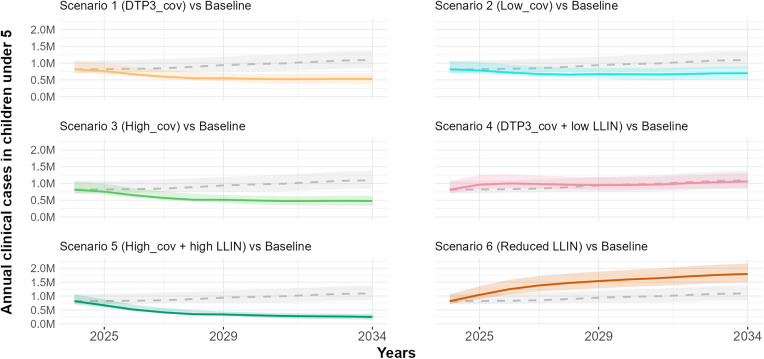
Comparison of annual clinical malaria cases projections under six scenarios with the baseline in CU5 (2024–2034). The baseline is represented by a dashed grey line with a shaded grey confidence interval, indicating the range of uncertainty (90% uncertainty interval). Scenario 1 (orange), Scenario 2 (cyan), Scenario 3 (green), Scenario 4 (pink), Scenario 5 (dark green), and Scenario 6 (dark orange) show projections under different intervention assumptions. The Baseline scenario assumes 0% vaccination coverage with 43.2% effective LLIN coverage. Scenario 1 assumes 76% vaccination coverage with 43.2% effective LLIN coverage; Scenario 2 assumes 50% vaccination coverage with 43.2% LLIN coverage; and Scenario 3 assumes 85% vaccination coverage with 43.2% effective LLIN coverage. Scenario 4 assumes 76% vaccination coverage with 28.8% effective LLIN coverage; Scenario 5 assumes 85% vaccination coverage with 50.4% LLIN coverage; and Scenario 6 assumes 0% vaccination coverage with 28.8% effective LLIN coverage.The solid lines represent the median estimates, while the shaded areas indicate the 90% uncertainty intervals (5th and 95th percentiles).

**Fig 7:**
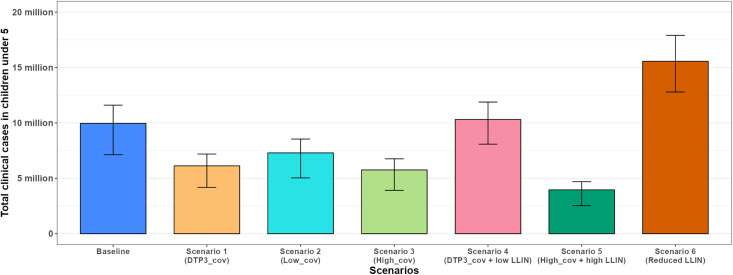
Total projected clinical cases in CU5 over 10 years (2025–2034). Bar graph showing the total malaria clinical cases in CU5 for the baseline (blue) and six other scenarios: Scenario 1 (orange), Scenario 2 (cyan), Scenario 3 (green), and Scenario 4 (pink), Scenario 5 (dark green), and Scenario 6 (dark orange). The bars represent the 90% uncertainty intervals (5th and 95th percentiles) for each scenario.

**Fig 8:**
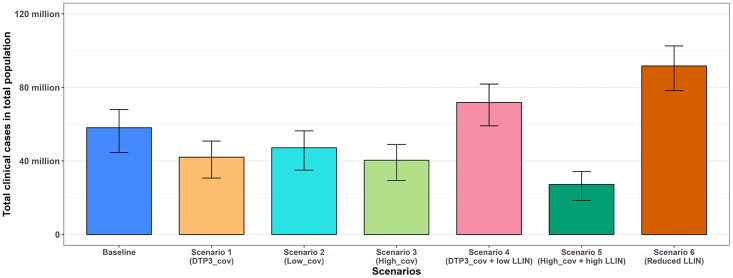
Total projected cases in the total population over 10 years (2025–2034). Bar graph showing the total clinical cases in the total for the baseline (blue) and six other scenarios: Scenario 1 (orange), Scenario 2 (cyan), Scenario 3 (green), and Scenario 4 (pink), Scenario 5 (dark green), and Scenario 6 (dark orange). The bars represent the 90% uncertainty intervals (5th and 95th percentiles) for each scenario.

In Scenario 5, which simulates the improvement of vaccine coverage and increase in effective LLIN coverage through SBCC, there is a substantial reduction of approximately 62% in clinical malaria cases in CU5 compared to the baseline, corresponding to a 2.86-fold reduction in all outcomes over the 10-year period. This reduction underscores the added benefit of enhanced intervention coverage, as Scenario 5 (85% vaccination + 50.4% effective LLIN coverage) has the potential to achieve approximately 1.7 times the reduction observed with Scenario 1 (76% vaccination + 43.2% effective LLIN coverage) in CU5. Moreover, Scenario 5 projected more cases and deaths averted across the entire population, attributable to an increase in effective LLIN coverage (Table 2–3, Figs 6–8, Figs G and J in S1 Appendix).

Scenario 6 detailing no vaccine introduction with a reduction in effective LLIN coverage (from 43.2% to 28.8%), projects an increase in clinical malaria cases, surpassing baseline levels in both CU5 and the total population. This scenario is designed to assess the relative contribution of vaccination by comparing outcomes with and without its addition under reduced effective LLIN coverage. Under this scenario, there is a substantial 36% increase in clinical malaria cases in CU5 (Table 2–3, Figs 6–7). This emphasizes that reducing effective LLIN coverage without introducing vaccination can lead to a worsening malaria burden as Scenario 6 has the potential to result in a large increase in malaria cases (36%), particularly in CU5 compared to Scenario 4 which results in only 4% increase due to the result that the vaccine benefits are partially masked by the reduced effective LLIN coverage.

This underlines the critical importance of both vaccination and LLINs in malaria control.

Figs 6 and 7 respectively illustrate the projected trajectories of annual clinical cases over time for all scenarios and the total projected clinical cases in CU5. Additionally, Fig 8 illustrates the total projected clinical cases in the total population. The total projected clinical cases in CU5 for each scenario influence the corresponding trends in severe cases and malaria-related deaths in CU5 (Figs H and I in S1 Appendix).

## Discussion

Based on a mathematical compartmental model of *P. falciparum* malaria, our study estimated the clinical cases and deaths averted in Benin, West Africa, following the introduction of the RTS,S malaria vaccine over a 10-year period (2025–2034). Considering six scenarios for vaccine coverage and LLIN use, this study projects that vaccine coverage at a national level of 85% in children, access to LLINs at 2020–2022 coverage levels and an increased use of LLINs at 70% across the country could lead to a 62% reduction in clinical malaria cases and deaths in children under five years of age. However, the introduction of the RTS,S malaria vaccine at DTP3 coverage levels (76%) to supplement the current use of nets (60%) was estimated to result in a substantial 40% reduction in clinical malaria cases and deaths in children under five. This suggests that vaccination can play a crucial role in reducing the malaria burden in children under five, even when LLIN use remains at current levels.

Our results qualitatively align with those reported in a modelling study by Hogan et al. [19], which estimated the impact of the RTS,S malaria vaccine allocation strategies in sub-Saharan Africa. The study revealed that in high-endemic areas, adopting vaccine coverage similar to that of DTP3 could prevent 4.3 million malaria cases and 22,000 deaths in children under five each year in sub-Saharan Africa. Moreover, our results qualitatively align with the study conducted by Hamilton et al. [7], which indicated that the widespread implementation of a malaria vaccine has the potential to decrease the malaria burden in Africa.

Scenario 4 was included in our simulation to project the impact of a decrease in net use on malaria transmission. When considered additively, the combination of vaccination and LLINs is projected to be effective in reducing malaria clinical cases. However, factors affecting their implementation must also be considered. The introduction of a malaria vaccine may negatively affect net use, which could lead to a relatively smaller reduction (or even an increase) in malaria burden [7]. In Scenario 4, the model projects that a 14.32% national decline in effective LLIN coverage compared to current levels, alongside vaccination, would result in an increase in malaria clinical cases and related deaths, surpassing baseline figures in the total population. Thus, even if the coverage (or distribution) of LLINs stays at the same average level as it was between 2020 and 2022, it is important to ensure that there is consistent net use. This can be promoted through SBCC.

To emphasize the importance of SBCC and to demonstrate its potential benefit for malaria prevention and control, Scenario 5 was included in our simulation. According to the previous three Benin DHS reports, there has been an increase in household population net accessibility (15% in 2006, 64% in 2011–2012, and 77% in 2017–2018) [26]. Similarly, the use of LLINs by household populations increased by 56% (from 14.7% in 2006 to 71.1% in 2018) [26]. However, the malaria indicators survey conducted in Benin in 2022 revealed that approximately 62% of the population use nets, lower than the 2018 reports [33]. Implementing high-quality strategic SBCC can enhance malaria prevention through campaigns aimed at raising awareness about the use of LLINs, the importance of seeking effective treatment, and other control measures [35].

In this regard, the primary behavioural objectives in Benin’s National Strategy for Malaria Social and Behavioural Change Communication 2021–2025 are to ensure that 90% of the population sleeps under LLINs every night, to promote early care-seeking and a 95% uptake of IPTp [3]. Given the critical role of SBCC in achieving these targets, we included Scenario 5 in our simulation to highlight its importance and potential impact on malaria prevention and control. Scenario 5, in which there is an increase in vaccine coverage compared to DTP3 levels and a 7.2% increase of the current effective LLIN coverage, resulted in a projected relative reduction of ~62% in all outcomes compared to baseline. While our study highlights the potential impact of SBCC and increased LLIN coverage on malaria control in Benin, it is crucial to consider the evolving challenge of insecticide resistance. Resistance can reduce the effectiveness of LLINs by allowing mosquitoes to survive contact with treated nets. Although LLINs continue to offer individual protection even in areas with high resistance [36], the emergence of resistance can lead to increased malaria transmission [37]. This underscores the importance of integrating alternative vector control strategies, such as next-generation nets alongside SBCC efforts to sustain and enhance malaria prevention outcomes [38].

In addition to LLIN intervention, malaria vaccination combined with other control options, such as SMC, could potentially reduce the malaria burden beyond our current projections. Several studies have evaluated the combination of malaria vaccination with chemoprevention and suggested this combination as a viable malaria control method. Chandramohan et al. [39], through an individually randomized controlled trial, revealed that the combination of vaccination and SMC resulted in a significant decrease in the incidence of malaria cases and deaths compared to implementing either intervention alone, highlighting the additional benefits of their combined use. Moreover, a mathematical modelling study demonstrated that adding seasonally targeted RTS,S to SMC would result in a greater reduction in clinical incidence compared to using SMC alone [40]. However, it is important to acknowledge that cost considerations will play a critical role in policymaking. The implementation of RTS,S entails vaccine procurement, delivery, and programmatic costs, while SBCC strategies to increase LLIN use generally involve lower unit costs but require sustained investment to maintain behaviour change [41–43]. Previous economic evaluations in malaria-endemic settings have highlighted that both vaccination and SBCC can be cost-effective depending on context [42,44]. Therefore, future research should prioritize evaluating the impact and cost-effectiveness of combining the RTS,S malaria vaccine with SMC, as well as incorporating SBCC to increase LLIN use, while considering variations in seasonality in the Benin context. Such studies are crucial for guiding policy decisions and optimizing resource allocation to maximize the reduction of malaria burden in children.

To the best of our knowledge this is the first national-level mathematical modelling study to estimate the impact of the RTS,S/AS01 malaria vaccine on malaria incidence in Benin. The findings of this study can provide valuable insights for policymakers and contribute to the growing body of evidence on the impact of RTS,S on malaria transmission in Benin.

Our study has several limitations related to the assumptions of our compartmental model and the data used for calibration. Our model used WMR estimates of cases and deaths as the basis for calibration. While these provide standardized country-level estimates, they may not fully capture local realities, which could affect the precision of vaccine impact projections. Moreover, our use of a country-level approach assumes the same incidence, vaccine coverage, and nets use at a national level. A detailed study at a sub-national level calibrated to local data is required to provide estimates of the vaccine impact at a sub-national level. We considered the primary series (three doses) of the RTS,S/AS01 malaria vaccine. A fourth dose or any subsequent doses up to five doses delivered annually could potentially reduce the disease burden further than our projections. We considered the same coverage for LLIN use among both children and adults, but children are more likely to use nets than adults. Our approach was based on the malaria indicator survey, which indicated approximately 60% LLIN use (in both children and adults) in 2022, while the SBCC strategy aims to achieve 90% of the population sleeping under nets. These structural and parameterization assumptions may influence our estimates of vaccine and LLIN impact. Future research should consider these limitations to provide a more accurate assessment of the impact of vaccination and LLIN use in children. That said, our framework does not perfectly represent the real-world complexity of malaria transmission but rather provides a scenario-based analysis of possible outcomes.

## Conclusion

This study employed a compartmental model to estimate the reduction in clinical malaria cases achieved by combining the RTS,S/AS01 vaccine with LLINs in Benin. Adopting childhood malaria vaccination at a coverage similar to the national DTP3 coverage levels, combined with the current use of long-lasting insecticidal nets, is projected to result in a greater reduction in clinical malaria cases and deaths among children under five years old compared to using nets alone. However, the vaccine must be combined with a consistent distribution and use of nets to achieve substantial malaria burden alleviation, highlighting the need for continued relationships with donors and increased domestic investment in the context of reduced international support.

## Supporting information

S1 Appendix Model description and equations. (DOCX)

S1 Table WHO reported annual malaria cases versus model predictions. (XLSX)

S1 Data Nets distribution from WHO. (XLSX)

S2 Table Sensitivity analysis results. (XLSX)

S2 Data Model output of annual malaria cases under baseline and intervention scenarios, disaggregated by year and age group. (XLSX)

S3 Data Model-generated cumulative malaria cases, comparing baseline and intervention scenarios across the simulation period. (XLSX)
